# Antifungal Activity of *Melaleuca alternifolia* Essential Oil (TTO) and Its Synergy with Itraconazole or Ketoconazole against *Trichophyton rubrum*

**DOI:** 10.3390/molecules26020461

**Published:** 2021-01-17

**Authors:** Janira Roana, Narcisa Mandras, Daniela Scalas, Paolo Campagna, Vivian Tullio

**Affiliations:** 1 Department of Public Health and Pediatrics, Microbiology Division, University of Turin, via Santena 9, 10126 Turin, Italy; janira.roana@unito.it (J.R.); narcisa.mandras@unito.it (N.M.); 2 Department of Veterinary Sciences, University of Turin, Grugliasco, 10095 Turin, Italy; daniela.scalas@unito.it; 3 Società Italiana per la Ricerca Sugli Oli Essenziali, 00161 Rome, Italy; paolocampagna51@gmail.com

**Keywords:** *Trichophyton rubrum*, essential oils, tea tree oil, *Melaleuca alternifolia*, azoles, checkerboard, synergism

## Abstract

Over the past 20–30 years, *Trichophyton rubrum* represented the most widespread dermatophyte with a prevalence accounting for 70% of dermatophytosis. The treatment for cutaneous infections caused by *Trichophyton* spp. are imidazoles (ketoconazole (KTZ)) and triazoles (itraconazole (ITZ)). *T. rubrum* can develop resistance to azoles after prolonged exposure to subinhibitory concentrations resulting in therapeutic failures and chronic infections. These problems have stimulated the search for therapeutic alternatives, including essential oils, and their potential use in combination with conventional antifungals. The purpose of this study was to evaluate the antifungal activity of tea tree oil (TTO) (*Melaleuca alternifolia* essential oil) and the main components against *T. rubrum* and to assess whether TTO in association with KTZ/ITZ as reference drugs improves the antifungal activity of these drugs. We used a terpinen-4-ol chemotype (35.88%) TTO, and its antifungal properties were evaluated by minimum inhibitory and minimum fungicidal concentrations in accordance with the CLSI guidelines. The interaction between TTO and azoles was evaluated through the checkerboard and isobologram methods. The results demonstrated both the fungicide activity of TTO on *T. rubrum* and the synergism when it was used in combination with azoles. Therefore, this mixture may reduce the minimum effective dose of azole required and minimize the side effects of the therapy. Synergy activity offered a promise for combination topical treatment for superficial mycoses.

## 1. Introduction

Dermatophytes are the most common fungal agents leading to superficial skin infections. During the last century, their spread changed under the influence of several elements, such as socioeconomic factors, practices bound to life-style, increasing travel, and migration from southern to northern countries and vice versa [1]. *Trichophyton rubrum*, frequently causing *tinea pedis*, *tinea unguium*, and *tinea corporis*, has increased steadily over this period and became the main agent isolated worldwide [2,3,4]. Over the past 20–30 years, *T. rubrum* has represented the most widespread dermatophyte in Central and North Europe with a prevalence accounting for about 70% of dermatophytosis. This species is often the key etiological agent of chronic infections with a slow progression, suggesting that the fungus has adapted to the human host [5]. Sometimes it is responsible for a deeper and disseminated infection, particularly in immunocompromised patients [3].

**Figure 1:**
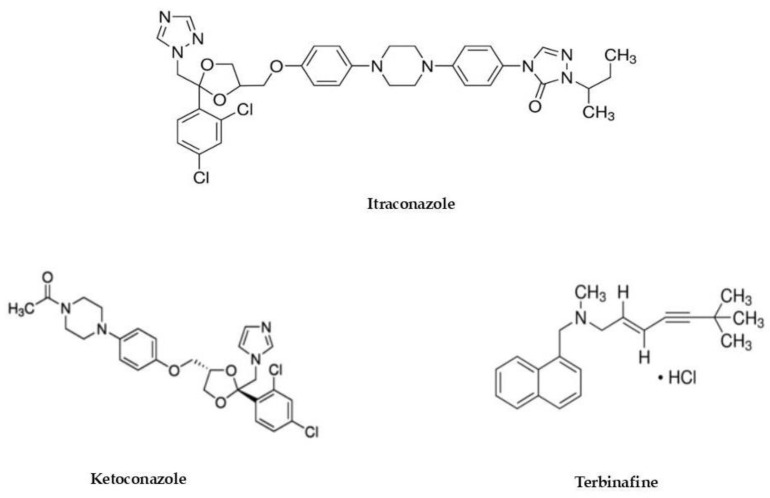
Chemical structures of azoles (itraconazole, ketoconazole) and terbinafine.

Azoles and allylamines (such as terbinafine) (Figure 1) are the most important oral and topical antifungal drugs used to treat dermatophytosis: they interfere with the ergosterol biosynthesis pathway causing structural modifications in the lipid membrane.

The preferred treatment for cutaneous infections caused by *Trichophyton* spp. are imidazoles, such as ketoconazole (KTZ), and triazoles, such as itraconazole (ITZ), often resulting in a complete clearance of the lesions. However, inappropriate prescribing, and the increased use of systemic antifungal agents in recent years are the main factors leading to the emergence of resistance to antifungal drugs (i.e., azoles) [6]. In fact, if not properly treated, dermatophytosis may become chronic, requiring oral antifungal drugs, which are often associated with side effects such as hepatotoxicity. *T. rubrum* can develop resistance to azoles after prolonged exposure to subinhibitory concentrations of these drugs leading to therapeutic failures and to the persistence and chronicity of the infections. Furthermore, complications like bacterial superinfections can occur [7].

These problems have stimulated the search for therapeutic alternatives, including natural compounds such as essential oils, which have been increasingly used in recent years for their biological and antimicrobial activities. Furthermore, a recent focus of research on essential oils is to investigate their potential use in combination with conventional antimicrobial drugs [8,9]. In fact, essential oils are one of the most evident promising sources for pharmacological research and the development of new natural antimicrobial agents against many microbial pathogens such as bacteria, yeasts, filamentous fungi, including dermatophytes, and viruses [10]. Among the best known oils, tea tree oil (TTO), the essential oil of *Melaleuca alternifolia* (Myrtaceae), often used as an antiseptic to treat skin infections, has recently received much attention for its antifungal properties. Because of its lipophilic nature, which facilitates skin penetration, it is used as a local formulation for dermatological disorders [11].

Hence, the purpose of this study was to evaluate the antifungal activity of TTO and its main components against *T. rubrum* and assess for the first time whether TTO in association with ITZ or KTZ improves the antifungal activity of these drugs against *T. rubrum*.

## 2. Results

### 2.1. TTO Composition

The phytochemical composition of TTO used for this study was determined by GC-MS, and the results are reported in Table 1.

TTO was found to be rich in terpinen-4-ol (35.88%) and γ-terpinene (19.65%), followed by a low percentage of α-terpinene (8.64%), p-cymene (4.61%), and 1,8-cineol (4.07%), in accordance with data from the ISO 4730:2017, as previously reported [11].

**Figure 2:**
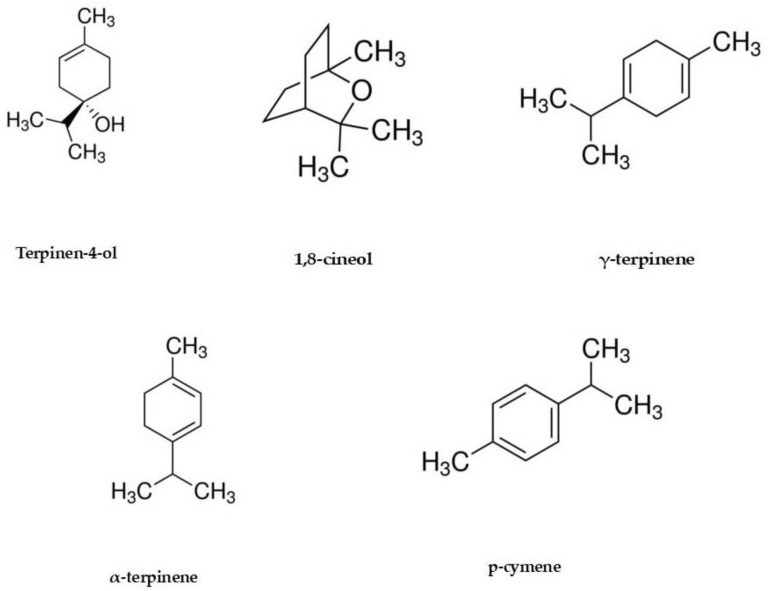
Chemical structures of the isolated compounds.

The chemical structures of the isolated compounds are reported in Figure 2.

### 2.2. Susceptibility Testing Results

On the basis of susceptibility testing results, the minimum inhibitory concentrations (MICs) did not differ significantly with regard to tested fungal strains: the mean MIC value obtained for TTO was 0.12% (*v*/*v*) (corresponding to 1.08 mg/mL), while for ITZ and KTZ as reference drugs was 0.5 µg/mL and 0.25 µg/mL, respectively. On the basis of minimum fungicidal concentration (MFC) results, TTO was found to display a fungicide activity against *T. rubrum* isolates (Table 2).

The two main components of TTO, terpinen-4-ol (35.88%) and γ-terpinene (19.65%), were also evaluated against the *T. rubrum* SL 171/13 strain (Table 2). The data revealed that terpinen-4-ol showed a lower MIC value (0.06%, *v*/*v*) than γ-terpinene (MIC = 0.5%, *v*/*v*) and TTO (MIC = 0.12%, *v*/*v*). (Table 2).

### 2.3. Checkerboard Assays and Assessment of FIC (fractional inhibitory concentration) Index

In order to determine what combination of TTO with azoles would be most effective, the checkerboard microtiter test was performed. The SL 171/13 strain was chosen because it demonstrated high sensitivity to TTO and both azoles. The combinations of TTO with the antifungal agents are reported in Table 3. By checkerboard testing, the binary associations of TTO with ITZ or KTZ were found to be synergistic against *T. rubrum* with FICI values of 0.245 and 0.37 (FICI < 0.5), respectively.

Moreover, TTO significantly potentiated the effect of azoles in combination therapy, reducing the MIC of both of them up to 8-fold: ITZ from 0.5 to 0.06 µg/mL and KTZ from 0.25 to 0.03 µg/mL. In addition, for each one of these interactions, the MIC for TTO (MICc) was below the testing concentration (MICa) (0.135/0.27 mg/mL vs. 1.08 mg/mL): it was remarkably decreased in the mixture with ITZ compared to the KTZ combination (8-fold vs. 4-fold).

**Figure 3:**
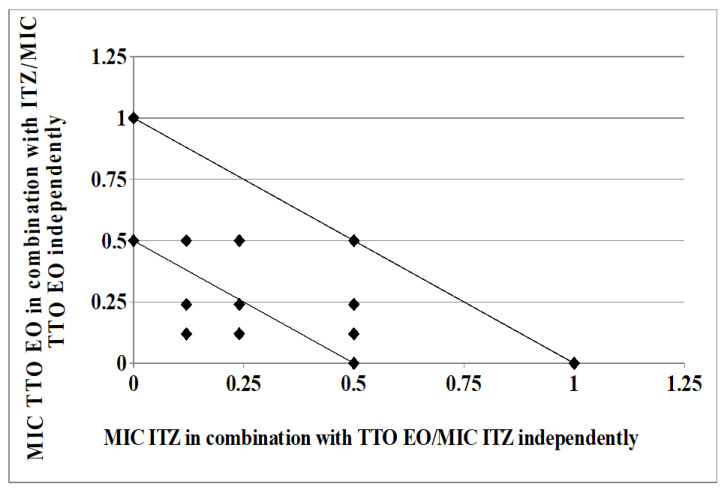
Isobologram of a synergistic interaction between TTO and ITZ against *Trichophyton rubrum* SL 171/13 strain. ITZ–FIC data are drafted on the *x*-axis, while TTO–FIC values are drafted on the *y*-axis.

**Figure 4:**
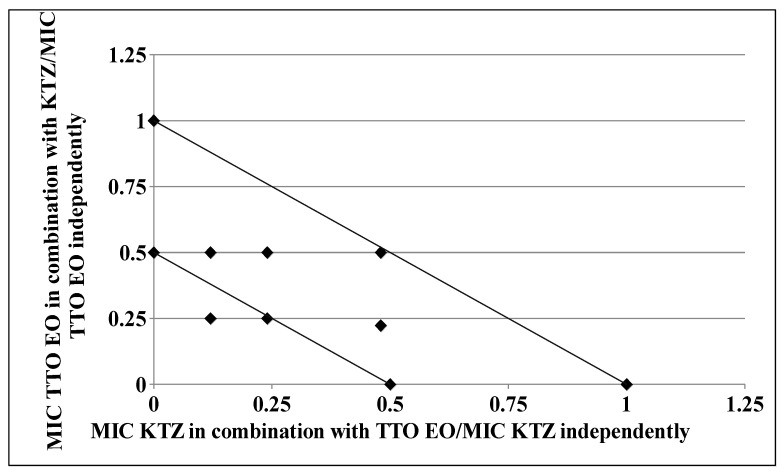
Isobologram of a synergistic interaction between TTO and KTZ against *Trichophyton rubrum* SL 171/13 strain. KTZ–FIC data are drafted on the *x*-axis, while TTO–FIC values are drafted on the *y*-axis.

As clearly depicted by the corresponding isobologram plots in Figure 3 and Figure 4, the presence of a synergistic association was confirmed by the plotted points moving towards the origin of the axes to produce a concave curve.

## 3. Discussion

Dermatophytoses have an unlikely spontaneous regression, and expensive, complex, and long-term therapy is often required. Antifungal drugs, such as azoles and terbinafine, play an important role in controlling these fungal infections but can have serious drawbacks, such as toxicity, fungistatic activity, and a limited spectrum of action or resistance [12]. In fact, dermatophytes with low sensitivity to antifungal drugs have occurred in many countries worldwide (i.e., Switzerland, Denmark, and India) [7]. Treatment of fungal infections caused by the drug-resistant fungi may require higher treatment doses. If azoles are used in higher doses in monotherapy, they can lead to adverse effects such as hepatotoxicity [13]. In addition, terbinafine-resistant *T. rubrum* isolates sometimes showed a low susceptibility to azole compounds: Monod et al. [7] showed that a strain from a male with *tinea pedis* was not responsive to standard therapy with terbinafine and ITZ. The great importance of natural products in the development of new therapeutic tools is becoming increasingly evident. In this respect, medicinal plants and their derivatives, such as essential oils, are important in phytotherapy, and for pharmacological research and drug development [14,15]. Moreover, essential oils represent a class of phytonutrients with potential antimicrobial and immunomodulatory capabilities [16].

TTO is an essential oil that has been used as a medicine since the beginning of the 20th century, when the Bundjalung aborigines of the northern coastal area of New South Wales (Australia) extracted TTO from the dried leaves of the *M. alternifolia* plant and used it for the treatment of superficial wounds. TTO is widely used in Australia, Europe, and North America for the treatment of *tinea pedis* and onychomycosis, mainly caused by *T. rubrum* and *T. mentagrophytes* in humans, and the treatment of dermatophytosis in horses caused by *T. equinum* [17,18]. TTO is also effective in inhibiting several fungal isolates, including *Candida albicans* and *Aspergillus niger*. *T. rubrum* is the main causative agent of almost all *tinea* infections worldwide, with the exception of *tinea capitis*, and it is likely to remain the dominant dermatophyte globally for the near future [19].

Natural compounds in combination with common antifungals can also minimize the side effects of the dose-related toxicity of these and contribute to the treatment of resistant microorganisms simultaneously [12].

Hence, the aim of this study was to assess for the first time whether TTO in association with ITZ or KTZ improves the antifungal activity of these azoles against *T. rubrum.* In this perspective, our findings highlight the synergistic interaction of TTO with ITZ or KTZ against a *T. rubrum* clinical strain. TTO also reduced the MIC of ITZ and KTZ up to 8-fold (from 0.5 to 0.06 µg/mL and 0.25 to 0.03 µg/mL, respectively). Moreover, another remarkable result is that the TTO MIC decreased in combination with azoles (0.135/0.27 mg/mL vs. 1.08 mg/mL) up to 8- and 4-fold (Table 2 and Table 3).

TTO is composed of terpene hydrocarbons, in particular monoterpenes, sesquiterpenes, and associated alcohols. The International Standard Organization (ISO 4730:2017) regulates the concentration ranges of the major TTO terpenes, related alcohols, and ethers [20]. Many types of commercial TTOs are known, according to ISO; however, most of the antimicrobial activity could be related to chemotypes containing 30–40% of terpinen-4-ol [21]. Hence, the effective antifungal activity of our chemotype against *T. rubrum* could be ascribed to terpinen-4-ol, accounting for 35.88% in the essential oil (Table 1). In fact, terpinen-4-ol was found to exhibit the strongest antifungal effect against dimorphic fungi and yeasts, such as *Saccharomyces cerevisiae* and *C. albicans.* [22]. Hydrophobic terpenes strongly interact with the membrane lipids of pathogenic microorganisms, thus affecting the permeability of the membrane. These alterations may lead to the degradation of the cell wall, reduction in the adherence to host surfaces, disruption of the plasma membrane, loss of cell content, coagulation of cytoplasm, and cell lysis. Moreover, terpinen-4-ol is able to affect, like azoles, the ergosterol biosynthesis of *S. cerevisiae*, leading to specific changes in gene expression [22].

Our data are in agreement with many studies that have demonstrated the synergistic or additive interactions of essential oils other than TTO with azoles, such as fluconazole and ketoconazole [8,9,13]. For example, Khan et al. reported synergistic interactions between oils of *Cinnamomum verum*, *Syzygium aromaticum*, *Cymbopogon martini*, *Thymus vulgaris*, and fluconazole against a multidrug resistant *T. rubrum* strain [13,23]. Moreover, Pyun et al. [24] observed significant synergistic antifungal activity when *Allium sativum* oil was combined with ketoconazole [24] against *Trichophyton* spp. (*T. rubrum*, *T. erinacei*, and *T. soudanense*). The synergistic combination of TTO with azoles may be explained by the essential oil promoting the effects of antifungal drugs, mainly on the cell wall, plasma membrane, and other membrane structures of *T. rubrum*. In addition, it could be possible that the fungicidal effect of TTO lowers the levels of the drug required to exert antifungal actions. The essential oils damaging the cell wall and cell membrane may facilitate the azoles’ entry to the cell, leading to a greater effect on the ergosterol biosynthesis inhibition and adding to cell membrane destruction [8].

In this context, the mixture of antifungal drugs is necessary to reach a better therapeutic action against mycoses caused by dermatophytes [25,26], because of the advantage over monotherapy, consisting of a greater fungal killing mechanism and a decrease in the spread of resistant strains [9,10,11,13,27].

The results obtained have demonstrated both the effects of TTO on *T. rubrum* and the synergism when it was used in combination with azoles. Therefore, combining itraconazole or ketoconazole with TTO may reduce the minimum effective dose of azole required and thus minimize the side effects for the treatment of infections caused by *Trichophyton* species.

## 4. Materials and Methods

### 4.1. Essential Oil and Major Components

TTO (batch n. 140,208 year 2014), purchased from Primavera/Flora, Pisa, Italy as a distillation sample, was obtained from the fresh leaves of *M. alternifolia*. The essential oil composition was analyzed by GC-MS at Flora s.r.l. with a Clarus 500 gas chromatograph (Perkin Elmer, Milan, Italy). Terpinen-4-ol (CAS Number: 20126-76-5; ≥95.0% pure) (sum of enantiomers, GC) and γ-terpinene (CAS Number: 99-85-4; 97% pure) were purchased from Sigma-Aldrich (Milan, Italia, s.r.l.) and used as received without any further purification.

For the experimental assays, 100% essential oil and the major components were dissolved 1:10 in 100% dimethyl sulfoxide (DMSO; Sigma-Aldrich) and diluted in a RPMI-1640 medium with L-glutamine plus 0.2% glucose and without sodium bicarbonate (Sigma-Aldrich, Rome, Italy), as previously described [11]. Then, TTO solution was buffered to pH = 7 with 0.165 M morpholinepropanesulfonic acid (MOPS) (Sigma-Aldrich). The final TTO concentrations ranged from 4% to 0.0017% (*v*/*v*). Tween 80 (Sigma-Aldrich) (final concentration 0.001%, *v*/*v*) was used to enhance the essential oil solubility [11]. TTO was protected from light and humidity and maintained at 4 °C just before use [8,11].

### 4.2. Reference as Antifungal Agents

ITZ and KTZ powders (≥98% purity) were purchased from Sigma-Aldrich (n° I6657 and K1003, respectively). ITZ and KTZ stock solutions were made up in 100% DMSO (Sigma-Aldrich) and stored at −20 °C just before use.

### 4.3. Fungal Strains

Four clinical isolates of *T. rubrum* isolated from *tinea pedis* (SL 171/13, SL 160/13) and *tinea corporis* (SL 164/13, SL 136/13), kindly provided by Prof. Ornella Cervetti and Dr. Michele Panzone (Medical Sciences Department, University of Turin, Turin, Italy), were used. Strains were subcultured on Potato Dextrose Agar (PDA; Oxoid S.p.A., Milan, Italy) at 30 °C. After a week, aerial mycelium was collected with sterile swabs, transferred in a flask containing Nutrient Broth (NB; Oxoid S.p.A., Milan, Italy), incubated at 35 °C and swirled once each day to prevent conidiation by keeping the culture submerged. From these 3–5-day-old cultures, mycelium was fragmented using a Tissue Grinder Teflon^®^ Pestle (Thomas^®^ Scientific, Swedesboro, NJ, USA) and diluted in a RPMI 1640 (Sigma-Aldrich) broth medium to yield a final inoculum of 1.5 × 10^4^ cfu/mL [28].

### 4.4. Antifungal Susceptibility Testing

In vitro susceptibility testing assays of TTO and its components or azoles on four *T. rubrum* clinical isolates were performed using the CLSI M38-A2 broth microdilution method [29], except for the inoculum suspensions [27], with some modifications for TTO [10]. A volume of 100 μL of the inoculum suspension was transferred into microtiter plates containing 100 μL of two-fold serial dilutions of ITZ (range 0.0035–8 μg/mL), KTZ (range 0.0015–32 μg/mL), and TTO and/or components (range 0.006–4%), respectively. The RPMI 1640 medium alone and the RPMI-1640 containing DMSO were used as controls, with no inhibitory effect on fungal growth. ITZ and KTZ were used as reference drugs.

Microdilution plates were set up and incubated at 28–30 °C for 7 days. Minimum inhibitory concentrations (MICs) of azoles were determined visually as the lowest concentration of drug that produced a complete inhibition of growth relative to that of the growth control. MICs of TTO/components were read as the lowest concentration at which no fungal growth was observed. Minimum fungicidal concentrations (MFCs) were determined by spot inoculating 10 μL from wells with the inhibition of molds growth containing either azoles or TTO/components onto SDA plates, which were incubated at 35 °C for 72 h. The MFC was defined as the lowest concentration resulting in no growth on subculture [10].

### 4.5. Checkerboard Assays and Assessment of FIC Index

Combinatorial effects between ITZ/KTZ and TTO were evaluated by the checkerboard broth microdilution assay. A two-dimensional checkerboard with serial two-fold dilutions of each compound, ranging from several dilutions below the MIC to 2 × MIC, was set up. Binary combinations were mixed in a 96-well microtitre plate. Cell suspensions were added to each well containing binary mixtures of azole drug/TTO. Each strain was tested in duplicate. Plates were incubated by shaking (150 rpm) at 28–30 °C for 7 days, and, afterwards, they were read visually. The results were analyzed using the fractional inhibitory concentration index (FICI) which was calculated as follows: FICI = FICa + FICb = MICa in combination/MICa tested alone + MICb in combination/MICb tested alone; where MICa and MICb are the MICs of azole and of the tested essential oil used alone. Synergy and antagonism were defined by FICI values of ≤0.5 and >4, respectively. A FICI value between 0.5 and 1.0 was considered as additive, while a value between 1.0 and 4.0 was considered as indifferent [8,9,16,30].

### 4.6. Isobolograms

The results of the checkerboard assays were represented graphically by isobolograms, as previously described [8,30]. FIC values along the growth-no growth interface were calculated and reported by plotting FIC values of drug along the ordinate, and FIC values of TTO along the abscissa. The straight line that joins the intercept points is the line of additivity (FICI = 1). Below this line, we find the area of additive (0.5 < FICI ≤ 1) and synergistic (FICI ≤ 0.5) effects, respectively. FIC index values above of the straight line were interpreted as corresponding to indifferent (1 < FICI < 4) or antagonistic (FICI > 4) interactions [30].

### 4.7. Data Analysis

The results were obtained from three independent experiments performed in triplicate and expressed as modal results.

## 5. Conclusions

The limited range of conventional and effective antifungal agents against *Trichophyton* spp. and the possible appearance of unpleasant adverse effects on the host are linked to safety problems and have lead to the search for a new, effective broad-spectrum combination of drug and natural antifungal. It is known that *T. rubrum* exhibited a relatively higher production of virulence factors, such as keratinase and elastase, which can degrade the skin structural barrier and immune cells [23,31]. The synergy between drugs and essential oils could allow the use of lower doses of azoles in an effective and safe way, offering a promising use of this combination with TTO in the future pharmaceutical preparation for cutaneous mycoses management. In fact, some studies [23,32] showed that certain essentials oils and their components (*Thymus vulgaris*, thymol, *C.copticum*, and *Origanum vulgare*) are able to inhibit elastase in *T. rubrum* [32].

However, the evaluation of the safety and therapeutic efficacy of the essential oil in combination with antifungal drugs needs further in vitro and in vivo experiments.

## Figures and Tables

**Table 1:** Phytochemical composition of *Melaleuca alternifolia* essential oil (tea tree oil (TTO)).

Main Components	*Melaleuca alternifolia* EO (%)
Terpinen-4-ol	35.88
1,8-cineol	4.07
γ-terpinene	19.65
α-terpinene	8.64
p-cymene	4.61

**Table 2:** MIC ^1^ and MFC ^2^ of Tea Tree Oil (TTO) and azoles (used as reference drugs) against *Trichophyton rubrum* strains.

T. rubrum Isolates		TTO (% *v*/*v*)	ITZ (µg/mL)	KTZ (µg/mL)
SL 171/13	MIC	0.12	0.5	0.25
	MFC	0.12	0.5	0.25–0.5
SL 164/13	MIC	0.06/0.12	0.5	0.25
	MFC	0.06/0.12	0.5	0.25–0.5
SL 160/13	MIC	0.06	0.25–0.5	0.25
	MFC	0.06/0.12	0.25–0.5	0.25
SL 136/13	MIC	0.12	0.5	0.25
	MFC	0.12	0.5	0.5
			**terpinen-4-ol ^3^** **(% *v*/*v*)**	**γ terpinene ^3^** **(% *v*/*v*)**
SL 171/13	MIC		0.06	0.5
	MFC		0.12	0.5

^1^ MIC = minimum inhibitory concentration; ^2^ MFC = minimum fungicidal concentration; ^3^ Main TTO components; Bold: separate the oil and drugs data from the components

**Table 3:** MIC ^1^ and MFC ^2^ indexes of Tea Tree Oil (TTO) plus itraconazole (ITZ) or ketoconazole (KTZ) against *Trichophyton rubrum* SL 171/13 strain.

	MICa	MICc	FIC ^3^	FICI ^4^	Interpretation
TTO-ITZ					
TTO (mg/mL) (% *v*/*v*)	1.08 ^5^ (0.12%)	0.135 (0.015%)	0.125	0.245	SYNERGY
ITZ (µg/mL)	0.5	0.06	0.12		
					
TTO-KTZ					
TTO (mg/mL) (% *v*/*v*)	1.08 ^5^ (0.12%)	0.27 (0.03%)	0.25	**0.37**	SYNERGY
KTZ (µg/mL)	0.25	0.03	0.12		

^1^ MIC = minimum inhibitory concentration; ^2^ MFC = minimum fungicidal concentration; ^3^ FIC =  MIC of azole (or TTO) in combination/MIC of azole (or TTO) alone; ^4^ FICI (FIC Index)  =  FIC of azole + FIC of TTO. FICI < 0.5: synergy. ^5^ MIC expressed as mg/mL.

## Data Availability

The data presented in this study are available on request from the first author Janira Roana. The data are not publicly available due to privacy.

## Sample Availability

Samples of the compounds TTO, itraconazole, ketoconazole, and dermatophytes (*Trichophyton rubrum*) are available from the authors.
